# Identification of protein lysine methylation readers with a yeast three-hybrid approach

**DOI:** 10.1186/s13072-018-0175-3

**Published:** 2018-01-25

**Authors:** Agnieszka Anna Rawłuszko-Wieczorek, Franziska Knodel, Raluca Tamas, Arunkumar Dhayalan, Albert Jeltsch

**Affiliations:** 1 Department of Biochemistry, Institute of Biochemistry and Technical Biochemistry, Stuttgart University, Allmandring 31, 70569 Stuttgart, Germany; 2 Department of Biochemistry and Molecular Biology, Poznań University of Medical Sciences, Święcickiego 6 St., 60-781 Poznan, Poland; 3 Department of Biotechnology, Pondicherry University, R.V. Nagar, Kalapet, Pondicherry, 605014 India

**Keywords:** Lysine methylation, Readers of posttranslational modifications, H3K9me3

## Abstract

**Background:**

Protein posttranslational modifications (PTMs) occur broadly in the human proteome, and their biological outcome is often mediated indirectly by reader proteins that specifically bind to modified proteins and trigger downstream effects. Particularly, many lysine methylation sites among histone and nonhistone proteins have been characterized; however, the list of readers associated with them is incomplete.

**Results:**

This study introduces a modified yeast three-hybrid system (Y3H) to screen for methyllysine readers. A lysine methyltransferase is expressed together with its target protein or protein domain functioning as bait, and a human cDNA library serves as prey. Proof of principle was established using H3K9me3 as a bait and known H3K9me3 readers like the chromodomains of CBX1 or MPP8 as prey. We next conducted an unbiased screen using a library composed of human-specific open reading frames. It led to the identification of already known lysine methylation-dependent readers and of novel methyllysine reader candidates, which were further confirmed by co-localization with H3K9me3 in human cell nuclei.

**Conclusions:**

Our approach introduces a cost-effective method for screening reading domains binding to histone and nonhistone proteins which is not limited by expression levels of the candidate reading proteins. Identification of already known and novel H3K9me3 readers proofs the power of the Y3H assay which will allow for proteome-wide screens of PTM readers.

**Electronic supplementary material:**

The online version of this article (10.1186/s13072-018-0175-3) contains supplementary material, which is available to authorized users.

## Background

Posttranslational modifications (PTMs) of proteins are utilized by eukaryotic cells to structurally and functionally diversify the proteome. Protein methylation is one of the most widespread PTMs involved in the majority of cellular processes and signaling pathways. Protein methyltransferases modify the side chains of lysine, arginine, histidine, or glutamine residues in proteins, with a prevalence of lysine and arginine methylation. In addition, the N- and C-termini of proteins can be methylated [1, 2]. During the last decade, the discovery of the prominent roles of lysine methylation in histone tails intensified studies on this PTM [3, 4]. Different lysine residues can be subjected to modification by one, two, or three methyl groups at their ε-amine group, and each methylation state potentially has distinct functions in regulating transcription and chromatin compaction [3, 5]. Importantly, methylation does not substantially alter the biophysical properties of the lysine side chain, but it serves as a docking site for binding proteins called ‘readers’ that exert their function on chromatin [4, 6]. Depending on the methylation level and location of the modified residue, ‘readers’ trigger various effects including, but not limited to, regulation of chromatin compaction, recruiting other chromatin complexes or factors involved in DNA metabolism. For example, genomic regions tri-methylated at H3K9 or H3K27 are usually silenced, whereas tri-methylation at H3K4 characterizes active promoters [6, 7]. Hence, deciphering the molecular basis of PTM signaling requires knowledge about the modification enzymes, but also the reading domains. Over last almost 2 decades, more than fifty lysine-specific methyltransferases (PKMT) have been identified [6, 8] which inspired follow-up discoveries of methyllysine readers. Moreover, histone methylation studies indicated that PKMTs are also involved in the modification of nonhistone proteins [1, 9]. Subsequently, some histone methylation readers were shown to act on nonhistone proteins as well [10, 11], but in general, readers of nonhistone protein lysine methylation are rarely known. Hence, even though a number of histone and nonhistone protein lysine methylation readers have been characterized, the list is unlikely to be complete.

Over last years, various approaches based on protein arrays, peptide arrays or stable isotope labeling (SILAC) and modification specific pull down were developed to identify methyllysine readers [12, 13]. However, these methods rely on the optimal expression of readers in the specific cell type under the tested conditions or require the expression and purification of reader candidates in functional form. Considering the fundamental role of lysine methylation in cellular processes, there is an urgent need to develop new, sensitive techniques for the discovery of methyllysine readers. Here, we present a modified yeast three-hybrid (Y3H) system able to identify methylation-dependent protein–protein interactions. We demonstrate the proof of principle of the method, and, more importantly, we show that an unbiased screen using a library composed of human-specific open reading frames led to the identification of already known lysine methylation-dependent readers and novel methyllysine reader candidates.

## Methods

### Yeast plasmids design and molecular cloning

**Fig. 1:**
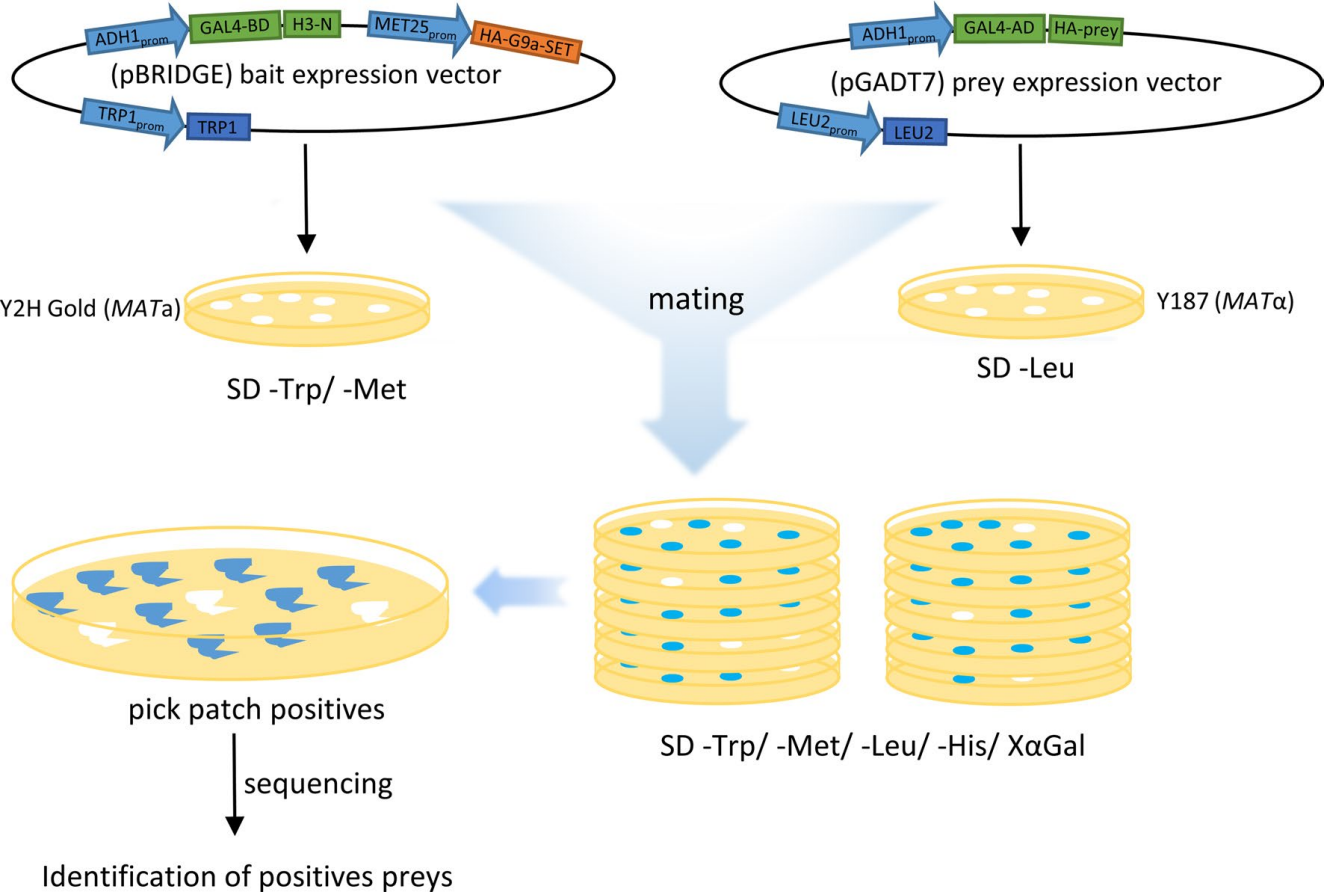
Schematic workflow of the Y3H experiment. The bait plasmid is composed of a GAL4-binding domain (GAL4-BD) fused with a histone 3 amino acids 1–20 (H3N) possessing the amino acid sequence that can be methylated at H3K9 by the HA-tagged catalytic domain of G9a enzyme (HA-G9a-SET). The prey plasmid is expressed as a fusion of GAL4-activation domain (GAL4-AD) and HA-tagged known reading domain of H3K9me3 (CBX1-CD, MPP8-CD) or human cDNA library. Haploid yeast strains transformed with pBRIDGE or pGADT7 expression plasmids were co-cultured to produce diploid yeast clones, expressing the bait and different prey proteins. The clone pool was plated on selective media to screen for interacting methylated bait with prey. Potential positive candidates were picked, patched, and grown further on selection media. Interacting preys were further isolated and the plasmids amplified in *E. coli* for sequencing of construct encoding region

Vectors used in this study were part of the Matchmaker Gold Yeast Two-Hybrid System (Takara Bio, Mountain View, CA, USA). The pBRIDGE vector was used as a backbone for the bait construct. To design a bait that will be recognized in a methylation-dependent manner, the catalytic SET domain of the G9a PKMT (G9a-SET; residues 931–1210) together with a nuclear localization sequence (NLS) and an HA-tag was cloned under the MET25 promoter in the MCS II region of the pBRIDGE vector using NotI and BglII restriction sites. On the same vector, the N-terminal twenty residues of histone H3 (referred later as an H3N) were cloned in fusion with the GAL4-binding domain (GAL4-BD) under an ADH1 promoter in the MCS I using EcoRI and BamHI restriction sites (Fig. 1). The bait plasmid with H3N located C-terminally with respect to GAL4-BD was also cloned. The pBRIDGE vector containing GAL4-BD-H3N but without G9a-SET was also generated as a negative control. The preys were expressed in the pGADT7 vector (Fig. 1). For the establishment of the system, the chromodomain of chromobox protein homolog 1 (CBX1-CD; residues 19–71) and chromodomain of M-phase phosphoprotein 8 (MPP8-CD; residues 58–112) were used. Both proteins were fused with the GAL4-activation domain (GAL4-AD) and HA-tag. All cloning procedures were performed by Gibson assembly [14]. The constructs were validated by sequencing. Screening for new H3K9me3 readers was performed using the Normalized Mate & Plate™ Universal Human cDNA Library as prey. The library pool constructed as GAL4-AD fusions cloned in pGADT7 and transformed in the yeast strain Y187 was purchased from the Clontech, cat. 630480 (Mountain View, USA).

### Yeast three-hybrid experiment

All experiments were performed in the yeast strains Y2H Gold (*MATa*) and Y187 (*MATα*), both purchased from the Clontech (Mountain View, USA). Yeast transformations were performed as described in Matchmaker Gold Yeast Two-Hybrid System Manual (Takara Bio, Mountain View, USA). Mating was performed by inoculation of the Y2H Gold yeast strain transformed with the bait constructs with the Y187 strain transformed with the prey constructs for 22–24 h. Diploids were then plated in 10× dilution series onto solid plates to assess reporter gene activation on nutrient deficiency medium SD-Trp/-Leu/-Met with 4 µg/ml of X-α-Gal for color change selection or SD-Trp/-Leu/-Met/-His with 4 µg/ml of X-α-Gal for color change and growth selection (Fig. 1). In library screens, one ml of Y187 pretransformed with the Mate & Plate Library was combined with four ml of Y2H Gold transformed with pBRIDGE-GAL4-BD-H3N-G9a and grown in 2xYPDA liquid medium containing 50 µg/ml kanamycin at 30 °C for 24 h with slow shaking (50 rpm). The diploids were plated on SD-Trp/-Leu and SD-Trp/-Leu/-Met/-His/X-α-Gal agar plates. All colonies from growth selection plates were transferred onto new plates to verify growth in the absence of His and emergence of blue color in the presence of X-α-Gal (Fig. 1). The inserts were identified using colony PCR with pGADT7 screening primers. The positive-interacting plasmids were isolated using Easy Yeast Plasmid Isolation Kit (Clontech, Mountain View, USA) and sent for Sanger sequencing (Fig. 1). Results were analyzed by comparison with the GenBank human database.

### Western blot analysis

Extraction of protein lysates was performed according to the Clontech Yeast Protocol Handbook. Briefly, 5 ml of overnight culture of bait and prey transformants in SD-Trp/-Met or SD-Leu medium was incubated in 50 ml of YPDA medium until OD_600_ reached 0.4–0.6. Yeast inoculates were spun down, and 0.44 ml of cracking buffer (8 M urea, 5% SDS, 40 mM Tris–HCl pH 6.8, 0.1 mM EDTA, bromophenol blue, β-mercaptoethanol and protease inhibitor) and 50 µl of glass beads (Cat # G8772; Sigma-Aldrich, St. Louis, USA) were added to total 33 units of OD_600_ of each pellet. Equal amounts of lysates were electrophoresed on a 16% SDS-PA gel and transferred to a nitrocellulose membrane. Membranes were blocked with TBST (10 mM Tris–HCl pH 7.5, 0.05% Tween 20, 150 mM NaCl) containing 5% skim milk for 1 h at RT, then washed three times with TBST, and incubated overnight at 4 °C with primary antibody anti-GAL4-BD (630403, Clontech), anti-HA.11 (16B12, BioLegend), or anti-phosphoglycerol kinase anti-PGK (22C5D8, Thermo Fisher) in 1% skim milk in TBST, all used in dilutions recommended by the supplier for western blot. After three washes with TBST, the membranes were incubated with proper HRP-conjugated secondary antibody anti-mouse (NXA931V, GE Healthcare) or binding protein m-IgGkappa BP-HRP (sc-516102, Santa Cruz Biotechnology) in 1% skim milk in TBST for 1 h at RT following another round of TBST washes. Bands were revealed using SuperSignal West Femto Chemiluminescent Substrate (Thermo Fisher Scientific, Waltham, USA).

### Cell lines

Irradiated mouse embryonic fibroblasts (iMEFs) wild type (WT) and *Suv39h1h2*^*−/−*^ were a gift of Prof. Thomas Jenuwein (MPI Freiburg). The cells were grown at 37  °C in Dulbecco’s modified Eagle’s medium and high glucose supplemented with 10% heat-inactivated calf serum, nonessential amino acids (Thermo Fisher Scientific, Waltham, USA), sodium pyruvate (Sigma-Aldrich, St. Louis, USA), 0.1 mM β-mercaptoethanol (Thermo Fisher Scientific, Waltham, USA) and 2 mM l-glutamine (Sigma-Aldrich, St. Louis, USA). Cells were grown at 37  °C in a saturated humidity atmosphere containing 5% CO_2_.

### Mammalian plasmids design and molecular cloning

The mVenus and mCerulean-C1 expression vectors were a gift from Prof. Steven Vogel (Addgene plasmids No. 27794 and No. 27796) [15]. The mCerulean-CBX1-CD construct was provided by C. Lungu as an H3K9me3 detector in iMEFs WT and *Suv39h1h2*^*−/−*^ [16]. The specific detection of H3K9me3 by mCerulean-CBX1-CD was validated by immunostaining with an H3K9me3 antibody (Additional file  1: Figure S1). The sequence encoding for the AGO3, HSFY1, ZNF470 and DCAF8 was amplified from HEK293 cDNA and assembled in mVenus vectors using the Gibson assembly procedure [14]. To maintain the biological context, constructs were not tagged with NLS. The constructs were validated by sequencing.

### Immunofluorescence and confocal microscopy

To determine the cellular localization of the selected candidate reader proteins in WT iMEF and *Suv39h1h2*^*−/−*^, cells were seeded on microscopy slides and transfected with the appropriate plasmids using Lipofectamine 3000 (Thermo Fisher Scientific, Waltham, USA). Forty-eight hours after transfection, the slides were washed with MgCl_2_ and CaCl_2_ containing PBS (Sigma-Aldrich, St. Louis, USA), followed by cross-linking with 4% formaldehyde solution (Sigma-Aldrich, St. Louis, USA) for 10 min at room temperature. After additional washing steps, the cells were mounted using Mowiol (Sigma-Aldrich, St. Louis, USA). The slides were imaged on an LSM 710 Zeiss confocal microscope equipped with a Plan-Apochromat 63×/1.40 Oil DIC M27 objective. The laser excitation wavelengths were 514 nm for mVenus and 405 nm for mCerulean. Emission collection windows were 525–602 nm for mVenus and 418–573 nm for mCerulean. Image analysis was performed in ImageJ. Control transfections confirmed the absence of cross talk between both channels. For analysis of the subnuclear localization of the fusion proteins, two categories were applied: spotty and homogenous. Categories were given based on spotty co-localization of a given construct with mCerulean-CBX1-CD or more homogenous localization of analyzed construct, while CBX1-CD preserved a spotty localization pattern of in iMEFs WT cells. The applicability of CBX1-CD construct for immunofluorescence detection of the H3K9me3 mark was previously demonstrated [16].

## Results

**Fig. 2:**
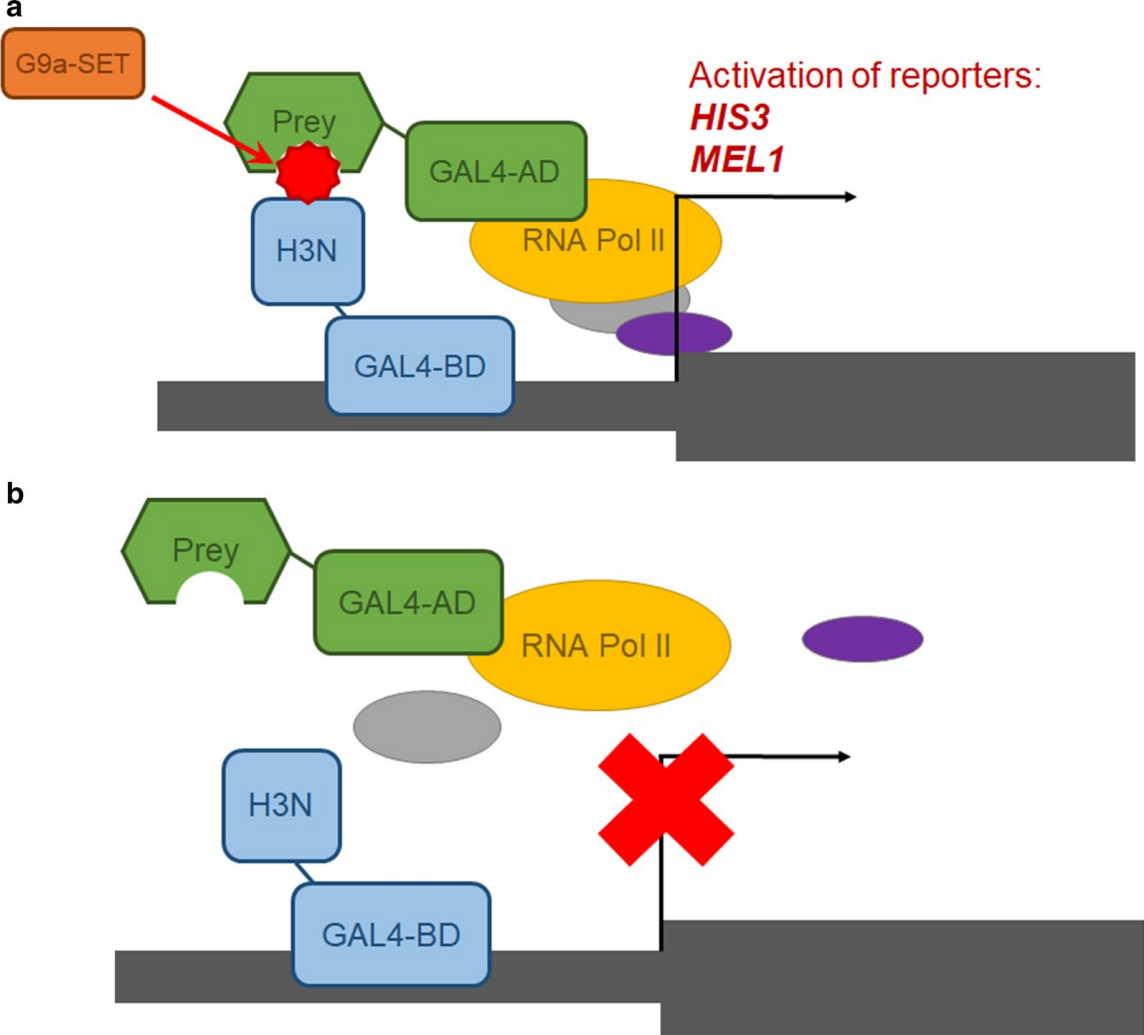
Principle of Y3H approach for detection of methylation readers. **a** Bait, GAL4-BD fused to H3 short polypeptide (H3N) recognizes and binds the promoter region of the reporter genes. The bait is methylated by the additionally expressed catalytic domain of G9a methyltransferase (G9a-SET). Interaction between methylated bait (H3K9me3) and prey recruits the GAL4-AD to the promoter upstream of the reporter genes, thereby reconstituting an active TF and as a result activating transcription of the *HIS3*-auxotrophic selection marker (which allows growth on media lacking histidine) and the *MEL1*-color selection marker (encodes α-galactosidase which in the presence of the chromagenic substrate X-α-Gal cause cells turn into blue). **b** In negative control settings, G9a-SET is not expressed, hence the bait is not modified and not recognized by the prey. As a result, GAL4 is not reconstituted, and the selection markers are not expressed

To identify novel reading domains able to specifically bind to lysine-methylated proteins, we developed a modified Y3H system, which expresses three proteins in yeast cells, viz. a bait, a PKMT that modifies the bait, and a prey responsible for recognition of the methylated bait. For proof of principle, we have used a short histone H3N-terminal tail construct (amino acids 1–20) as bait, as we aimed to screen for H3K9me3 methylation-specific readers. *Saccharomyces cerevisiae* is an especially well-suited cellular host for that system, because it lacks endogenous PKMTs that introduce this mark [17]. We have used the SET domain of G9a as PKMT that was shown to catalyze H3K9 tri-methylation in vitro and in vivo [18]. The H3 1–20 bait is sufficient to be methylated by G9a at K9 [10], but simultaneously excludes the side activity of G9a at H3K27. As a prey, we have adopted previously validated chromodomains of CBX1 and MPP8 able to bind H3K9me3. The crystal structures of MPP8-CD and CBX1-CD have been solved, and many biochemical assays confirmed their specific recognition of methylated H3K9 [19–21]. If the methylated bait interacts with the prey, the GAL4 transcription factor is reconstituted and triggers transcription of reporter genes (Fig. 2a). To verify the specificity of methylation-dependent recognition of the bait by selected preys, the system was also generated without G9a-SET. In this control setting, the prey should not bind the unmodified bait. Consequently, GAL4 should not be reconstituted indicated by a lack of color change and growth restriction (Fig. 2b).

### Bait and prey constructs were expressed correctly on selective media and presented no auto-activation and toxicity

**Fig. 3:**
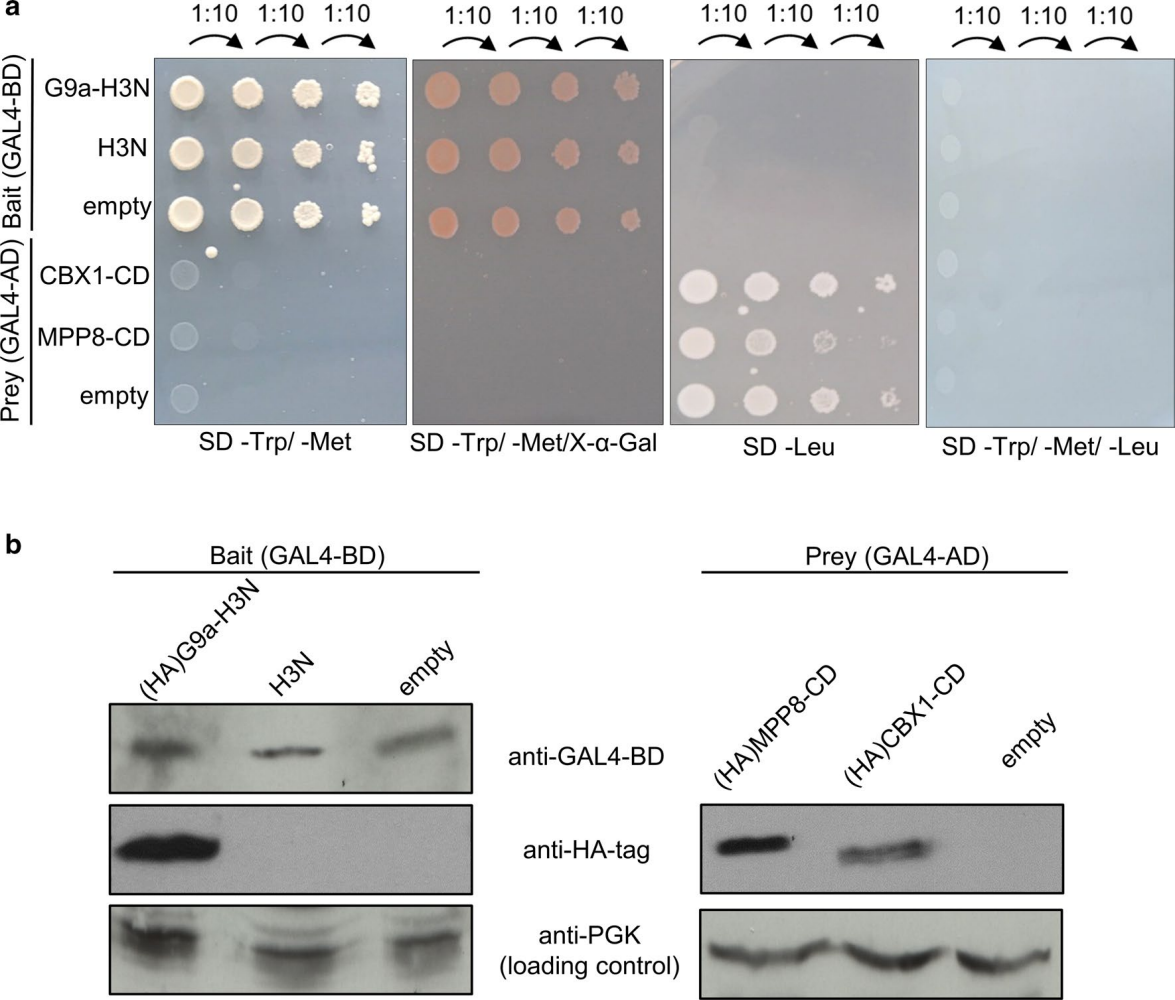
Expression of bait and prey constructs. **a** Yeast cells transformed with bait or prey were inoculated in liquid culture and spotted in serial dilutions onto SD-Trp/-Met, SD-Trp/-Met/X-α-Gal, SD-Leu, SD-Trp/-Met/-Leu plates. **b** Lysates from yeast expressing GAL4-BD or -AD were analyzed by western blot using antibodies to GAL4-BD, HA-tag, and PGK as protein loading control

Firstly, the expression of the bait and prey constructs in Y2H Gold and Y187, respectively, was validated on different selection media (Fig. 3a). The bait did not lead to autonomous activation of the reporter gene as shown by lack of color change on SD-Trp/X-α-Gal plate. Moreover, bait (GAL4-BD)-G9a-H3N or bait (GAL4-BD)-H3N yeast transformants seeded on the plates in serial dilutions indicated a lack of toxicity as shown by the absence of smaller colony size and similar growth as compared with empty bait control (Fig. 3a). Prey constructs (GAL4-AD)-CBX1-CD and -MPP8-CD also demonstrated growth on proper media and normal growth when compared with empty prey construct (Fig. 3a). Expression of all constructs was validated by western blot using antibodies against GAL4-BD or HA-tag to detect expression of bait, G9a-SET domain or prey constructs (Fig. 3b).

### Validation of the Y3H system for the identification of H3K9me3 readers

**Fig. 4:**
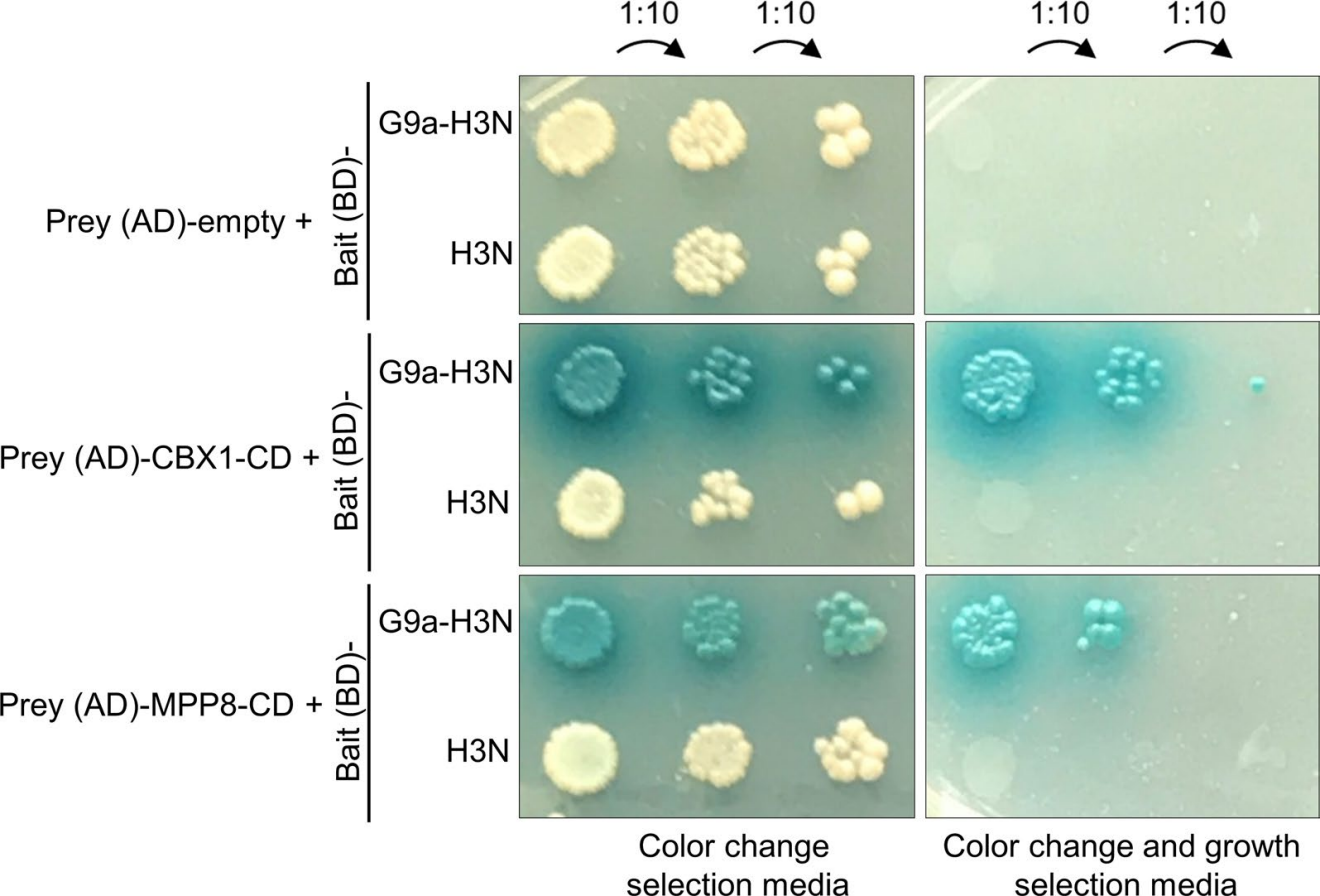
Methylation-dependent interaction of H3 (1–20) with chromodomain of CBX1 and MPP8 in yeast cells. Y2H Gold and Y187 yeast strain were transformed with bait (pBRIDGE-GAL4-BD) and prey (pGADT7-GAL4-AD) constructs, respectively. Transformed Y2H Gold cells were mated with Y187 harboring CBX1-CD, MPP8-CD in YPD-rich medium and plated in serial dilutions on selection media, either color change selection media (-Trp/-Leu/-Met/X-α-Gal) and color change and growth selection media (-Trp/-Leu/-Met/-His/X-α-Gal)

To investigate the potential use of the modified Y3H system for the discovery of new methylation readers, we first examined whether it can detect known H3K9me3 readers. The principle of the modified Y3H system is that the interaction of the methylated bait with the prey reconstitutes an active GAL4 transcription factor which then activates expression of two independent reporter genes. The *HIS3* gene is used as auxotrophic selection marker allowing for growth on media lacking histidine. The *MEL1* gene encodes a α-galactosidase, which in the presence of the chromogenic substrate X-α-Gal causes cells to develop a blue color and is used for color selection. Expression of the bait (GAL4-BD)-G9a-H3N or (GAL4-BD)-H3N in diploid cells together with the empty prey construct had no effect on colony color and preserved the inability to grow on selection media (Fig. 4). Haploid cells expressing the methylated bait were also mated with cells expressing preys CBX1-CD or MPP8-CD. In both diploid strains, activated expression of the reporter genes was observed by color change and growth on auxotrophic selection media (Fig. 4). In control settings lacking the methyltransferase (GAL4-BD)-H3N, no growth or color change was detected indicating the absence of interaction between the unmodified bait and prey (Fig. 4). These data clearly confirm the applicability of the modified Y3H system in the detection of methyllysine-dependent protein–protein interactions. A C-terminal location of H3 peptide to GAL4-BD was also tested, but resulted in significantly lower Y3H signals and was not used in further studies (data not shown).

### Identification of potential novel methylation-specific H3K9me3 readers

After demonstrating the applicability of our Y3H system, we performed a screen using a normalized human cDNA library fused with GAL4-AD with the methylated bait construct (GAL4-BD)-G9a-H3N to identify novel H3K9me3 readers. The mating resulted in 3.6 × 10^6^ diploid cells, which meet the demand for Y3H library screening. Out of them, around 200 colonies grew on selection media and were further investigated. The prey plasmids were isolated and retransformed into the Y187 yeast strain for another round of mating with Y2H Gold strain containing (GAL4-BD)-G9a-H3N or (GAL4-BD)-H3N to eliminate potential methylation-independent interactions. After elimination of false positives, self-activators, and duplicates, we identified 20 potential novel H3K9me3 readers and four  previsouly described H3K9me3 readers viz. (1) ATRX, (2) CBX1, (3) CBX5, and (4) MPP8 (Table 1). CBX1 and CBX5 are HP1 family members associated strongly with recognition of H3K9me3 and maintenance of heterochromatin at pericentromeric sites [22]. The crystal structure of MPP8-CD aromatic cage was solved and clearly indicates its binding to the methylated H3K9me3 [20, 23], whereas ATRX recognizes methylated H3K9 by its N-terminally located ADD domain [24]. The methylation-dependent interaction was confirmed with negative controls (bait without G9a-SET), where no growth or color change was observed. The identification of already known H3K9me3 readers in the library screen confirmed the functionality of the method.

**Table 1:** List of proteins identified in the Y3H screen

Protein name	Gene name	mRNA accession no.	Comment
ATRX, chromatin remodeler	*ATRX*	NM_000489.4	known H3K9me3 reader
Chromobox 1	*CBX1*	NM_001127228.1	known H3K9me3 reader
Chromobox 5	*CBX5*	NM_001127321.1	known H3K9me3 reader
M-phase phosphoprotein 8	*MPHOSPH8 (MPP8)*	NM_017520.3	known H3K9me3 reader
Argonaut 3	*AGO3*	NM_024852.3	H3K9me3 co-localization observed in fluorescence microscopy
DDB1 and CUL4 associated factor 8	*DCAF8*	NM_015726.3	H3K9me3 co-localization in observed fluorescence microscopy
Heat shock transcription factor, Y-linked 1	*HSFY1*	NM_033108.2	H3K9me3 co-localization in observed fluorescence microscopy
Zinc finger protein 470	*ZNF470*	NM_001001668.3	H3K9me3 co-localization in observed fluorescence microscopy
Ankyrin 3	*ANK3*	NM_001149.3	
Ankyrin repeat domain 7	*ANKRD7*	NM_019644.3	
ArfGAP with SH3 domain, ankyrin repeat and PH domain 1	*ASAP1*	NM_018482.3	
DEK proto-oncogene	*DEK*	NM_003472.3	
Essential meiotic structure-specific endonuclease 1	*EME1*	NM_001166131.1	
Family with sequence similarity 98 member A	*FAM98A*	NM_015475.4	
F-box and leucine-rich repeat protein 14	*FBXL14*	XM_017018875.1	
FERM and PDZ domain containing 1	*FRMPD1*	NM_014907.2	
Homeodomain interacting protein kinase 2	*HIPK2*	NM_022740.4	
Lamin B receptor	*LBR*	NM_002296.3	
Leiomodin 1	*LMOD1*	NM_012134.2	
SAM And SH3 domain containing 1	*SASH1*	NM_015278.4	
Sosondowah ankyrin repeat domain family member B	*SOWAHB*	NM_001029870.2	
Teashirt zinc finger homeobox 2	*TSHZ2*	NM_173485.5	
Zinc finger protein, FOG family member 2	*ZFPM2*	NM_012082.3	
Zinc finger protein 333	*ZNF333*	NM_001300912.1	

### Validation of H3K9me3 binding of novel candidate reading proteins in human cells

**Fig. 5:**
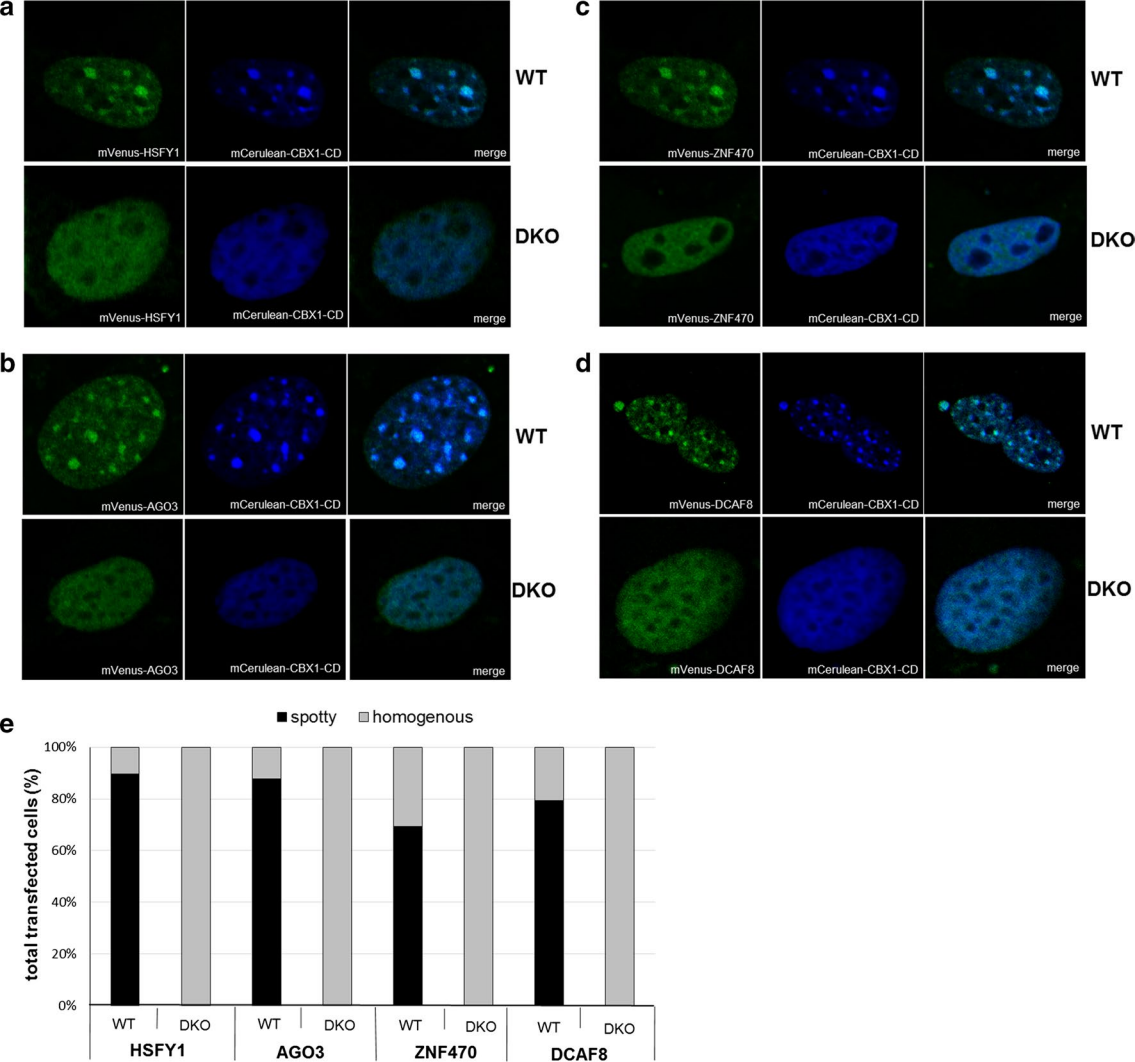
Representative fluorescence microscopy images documenting the co-localization of new H3K9 reading candidates with CBX1-CD. Results are shown for HSFY1 (**a**), AGO3 (**b**), ZNF470 (**c**) and DCAF8 (**d**) in iMEFs WT and *Suv39h1h2‒/‒* (DKO). The mVenus-tagged constructs co-localized with H3K9me3 detector mCerulean-CBX1-CD in iMEF-WT cells. The signal of mCerulean-CBX1-CD and analyzed mVenus-tagged constructs was homogenous in DKO cells. The transfection, imaging, and display settings of the images are identical. **e** Quantification of the biologically duplicated experiments representatively shown in **a**–**d**

Next, we turned our attention to novel potential H3K9me3 binders identified in the screen, including AGO3, HSFY1, DCAF8, and ZNF470 (Table 1). To address the question if these candidate proteins are H3K9me3 binders, we expressed them in iMEF wild-type cells (WT) and iMEF cells containing a double knockout of SUV39H1 and SUV39H2 (*Suv39h1h2*^*−/−*^), which do not contain pericentromeric H3K9me3 [25]. Mouse fibroblasts are a very useful system for the validation of H3K9me3 recognition, because mouse pericentric heterochromatin forms a punctate pattern that is easily identified in fluorescence microscopy. The H3K9me3 signal can, for example, be detected in cells with mCerulean-labeled CBX1-CD used in a previous study as an H3K9me3-specific detector [16]. In iMEFs WT cells, CBX1-CD forms spotty foci, whereas in *Suv39h1h2*^*−/−*^ cells the CBX1-CD subnuclear distribution is relatively homogenous, clearly lacking strong foci [16]. In our study, cells were transfected with mVenus-tagged reader candidates to investigate their potential co-localization with mCerulean-CBX1-CD (used as H3K9me3 marker). Upon transfection of mVenus-AGO3, DCAF8, HSFY1, and ZNF470, we observed highly significant overlaps of the distributions of the candidate readers and mCerulean-CBX1-CD in iMEFs WT cells (Fig. 5a–d). All experiments were performed in duplicates, and 25–40 cells were counted per biological replicate. In all constructs tested, co-localization with the H3K9me3 detector module (CBX1-CD) in iMEF-WT cells was seen for at least 70% of transfected cells (Fig. 5e). To verify H3K9me3 recognition of identified readers, the same experiment was performed in *Suv39h1h2*^*−/−*^ cells. Importantly, with all tested constructs we observed a homogenous distribution in the nucleus (Fig. 5a–d), indicating that the spotty phenotype was indeed dependent on the presence of heterochromatic H3K9me3. These observations confirm the results of the Y3H screen and suggest that the newly identified factors are indeed H3K9 methylation readers.

## Discussion

Lysine methylation of proteins directs and fine-tunes many cellular processes ranging from gene transcription, RNA processing to protein translation, and cell signaling [1]. Hence, understanding protein methylation readout is essential for elucidating fundamental mechanisms. The deciphering of the readout mechanism is based on understanding how the presence of a PTM is translated into a specific functional state. Often, PTMs serve as anchor point for binding of readers that trigger a physiological response. Here, we present a modified Y3H approach allowing to discover readers of H3K9me3. The method has been validated by the ability to identify already known H3K9me3 readers. In addition, we discovered and partially validated a set of interesting new interacting partners, AGO3, DCAF8, HSFY1, and ZNF470. Future studies will be needed to define the responsible reading domains and characterize the methylation-specific interaction with H3K9 with biophysical methods.

Available data already associate some of these newly identified reader candidates with H3K9 methylation. The short isoform of Argonaut 3, AGO3 also known as EIF2C3, belongs to the family of proteins which are involved in RNA interference. Recently, different groups independently confirmed RNA occupancy at pericentromeric regions and the RNA-mediated stabilization of the SUV39H1 methyltransferase at heterochromatin regions [26–28]. The detailed mechanism of pericentromeric H3K9 methylation in mammals is not precisely known, but in fission yeast, H3K9me3 is formed in an RNAi-dependent pathway [29, 30]. DCAF8 (WDR42A) belongs to the WD40 proteins family that are thought to function as receptors to selectively target different substrates and serve as scaffolds to facilitate the function and activity of other partners [31]. Recently, DCAF8 (WDR42A) was found as an essential partner of the Cullin 4-RING ligase complex (CRL4) [32]. The CRL4DCAF8 complex generates H3K79ub which in conjunction with increased H3K9me3 silences selected genes during liver development [32]. The connection of both modifications in this process is unknown. HSFY1 and ZNF470 are almost uncharacterized. ZNF470 belongs to zinc finger proteins possessing a Kruppel-associated box (KRAB) suggesting its potential function as a repressor [33].

The novel Y3H method complements already existing strategies to screen for PTM readers. Known strategies evolved from pull-downs of PTM containing proteins or peptides, followed by MS analysis [34] to more quantitative SILAC [35] or the BAC-GFP transgenomics technology [36]. However, MS-based approaches are still limited by their bias toward high-affinity interactions and by their inherent inability to detect interactors that are very weakly expressed or absent in the cell under investigation. The Y3H approach overcomes this limitation partially, because it uses a prey library representing all human ORFs. Methylation-specific detection is based on a binary interaction that does not rely on stoichiometry, cell-type specificity or external stimuli. Other approaches identifying PTM readers are protein arrays [12, 13], but this is experimentally challenging and limited in throughput.

One caveat of our approach is illustrated by the finding that while we were able to retrieve known interactors and suggest new ones, not all already known H3K9me3 binders were identified in the screen. This phenomenon might be related to common yeast hybrid system limitations like auto-activation of reporter genes by the prey proteins, prey toxicity, or inadequate protein folding upon expression in yeast, all leading to false negatives [37]. Still considering the outcome, the developed tool might be valuable as a low cost and the simple procedure that can detect direct interactors of methylated bait proteins.

The majority of previous studies focused on detection and readout of histone modifications, although the nonhistone protein methylation arises as a new center of interest. The main obstacle in the detection of PTM of nonhistones is their limited amount compared to histones. Although we developed the Y3H assay using H3K9 methylation readout as a test case, it can be theoretically applied to screen for nonhistone readers. Hence, this approach should allow discovering completely unknown readers of nonhistone PTM in an unbiased and binary way avoiding problems with the low physiological abundance of proteins. Moreover, a customized Y3H approach might also be a powerful tool to study the readout of methylation of other amino acid residues, including arginine, and of the N- and C-termini of proteins.

## Conclusions

Our approach for the identification of PTM readers complements other available methods and allows for an identification of direct binders of selected modification in an unbiased manner. It overcomes significant problems related to limitations of detection of weakly expressed or transient interactors and enables proteome-wide screening at relatively low costs. We identify novel H3K9 methylation reader candidates, the functions of which have to be studied in future. In principle, our method can also be applied to study nonhistone methylation readout, a massively underexplored field of research.

## Additional file

**Additional file 1: Figure S1.** Representative fluorescence microscopy images documenting the co-localization of CBX1-CD fluoresence and H3K9me3 antibody staining.

## Abbreviations

CBX1-CD: chromodomain of chromobox protein homolog 1
GAL4-AD: GAL4-activation domain
GAL4-BD: GAL4-binding domain
iMEF: irradiated mouse embryonic fibroblasts
MPP8-CD: chromodomain of M-phase phosphoprotein 8
NLS: nuclear localization sequence
PKMT: protein lysine-specific methyltransferase
PTMs: protein posttranslational modifications
SILAC: stable isotope labeling with amino acids in cell culture
Y3H: yeast-3-hybrid system
