# Supplementary material for: Identification of protein lysine methylation readers with a yeast three-hybrid approach
Source: Epigenetics Chromatin. 2018 Jan 25;11:4. doi: 10.1186/s13072-018-0175-3 (PMC5784651; doi:10.1186/s13072-018-0175-3)
Supplement: Supplementary file 1 — Additional file 1: Figure S1. Representative fluorescence microscopy images documenting the co-localization of CBX1-CD fluoresence and H3K9me3 antibody staining. [file 13072_2018_175_MOESM1_ESM.pdf]

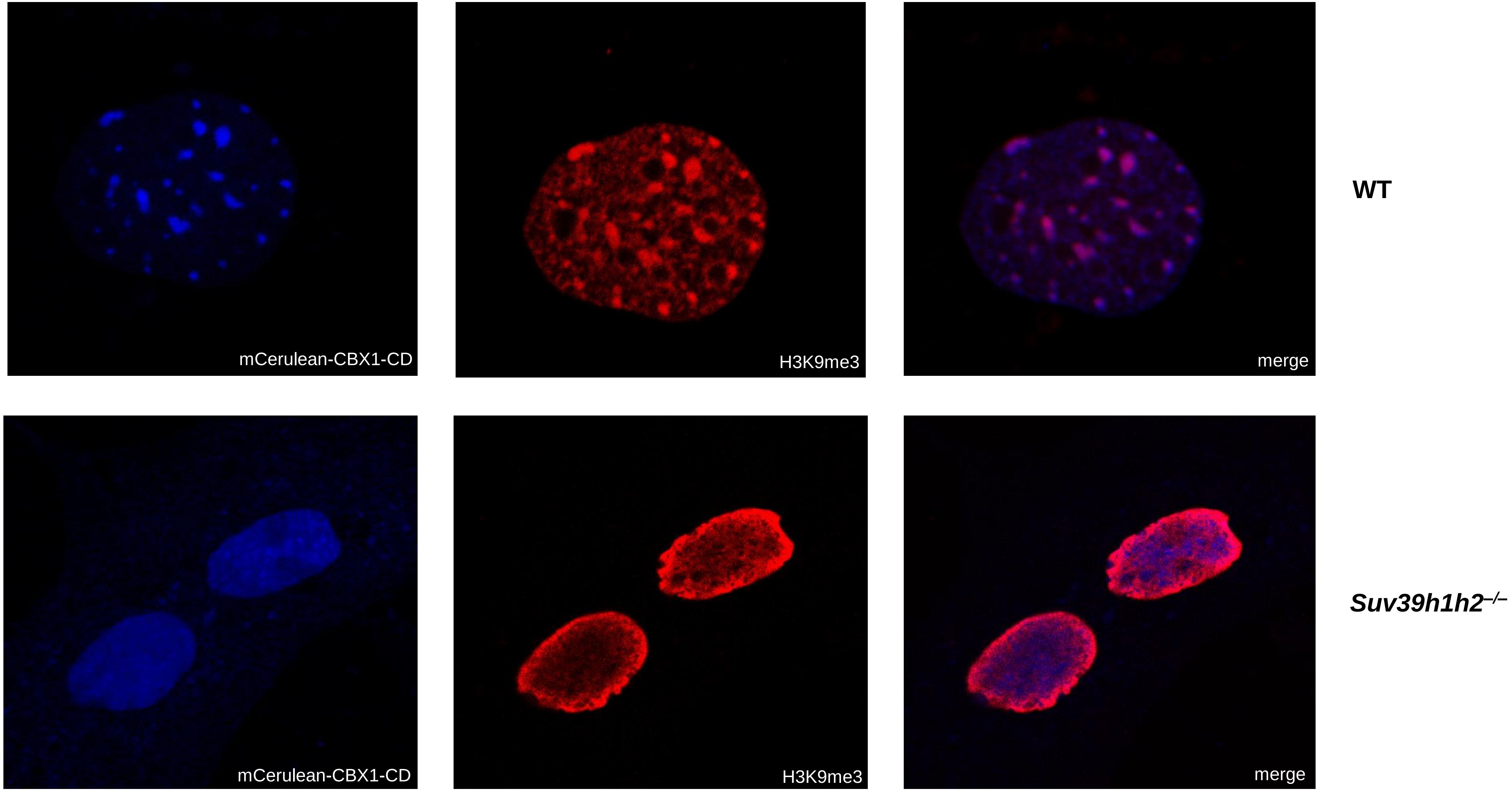

Figure S1. Representative fluorescence microscopy images documenting the co-localization of CBX1-CD fluoresence and H3K9me3 antibody staining. Results are shown for wildtype (WT) iMEF and *Suv39h1h2–/–* (DKO) cells. The mCerulean tagged CBX1-CD co-localized with H3K9me3 antibody in iMEF-WT cells. The signal of mCerulean-CBX1-CD and H3K9me3 antibody was homogenous in DKO cells. The imaging and display settings of the images are identical.
